# The Opium Disease in Infants and Children

**Published:** 1894-03

**Authors:** 


**The Opium Disease in Infants and Children.**—This was the subject of a paper by Dr. Louis Fischer, who expressed the belief that the disease was much commoner than was generally supposed; some cases diagnosticated as marasmus or as summer diarrhœa, were really cases of opium poisoning. Ignorant mothers and monthly nurses were generally responsible for the administration of the drug. One mother had bought opium of a Chinaman and given it to her child in half-grain doses. Another had administer as much as six tablespoonfuls of paregoric at a dose. The liability of children born of opium-eaters to die of collapse if the drug was withheld after birth was commented upon. The symptomatology included diarrhœa, sometimes with constipation, loss of appetite, irritability, restlessness, almost constant stupor, itching, as indicated by scratch marks, rapidity of the heart's action, decided sweating, scantiness of the urine, which was sometimes albuminous, and occasional vomiting—all this without fever. Besides sulphonal, which seemed to hold the first place in the treatment, brominated camphor, lupulin, chloral, and potassium bromide had been found useful. It was greatly to be deprecated that paregoric could be bought so readily without a prescription and without labels to indicate the doses proper for children of various ages.—*N. Y. Medical Journal.*

